# Hepatitis a among men who have sex with men in Barcelona, 1989-2010: insufficient control and need for new approaches

**DOI:** 10.1186/1471-2334-12-11

**Published:** 2012-01-20

**Authors:** Cecilia Tortajada, Patricia G de Olalla, Elia Diez, Rosa M Pinto, Albert Bosch, Unai Perez, Milagros Sanz, Joan A Caylà

**Affiliations:** 1 Epidemiology Department, Public Health Agency of Barcelona (ASPB), Pl. Lesseps,1, 08023 Barcelona, Spain; 2 CIBER Epidemiologia y Salud Pública (CIBERESP), Barcelona, Spain; 3 Preventive Intervention Department, Public Health Agency of Barcelona, Pl. Lesseps,1, 08023 Barcelona, Spain; 4 Enteric Virus Laboratory. Department of Microbiology, University of Barcelona, Av. Diagonal, 645, 08028 Barcelona, Spain

## Abstract

**Background:**

Men who have sex with men (MSM) are a known group at risk for hepatitis A and outbreaks among this group are frequent. In Barcelona, vaccination for MSM has been recommended since 1994. In 1998 a vaccination campaign among preadolescents was implemented and an immunization program in gay bathhouses began in 2004. Objective: to asses the incidence of hepatitis A in adults in Barcelona from 1989 to 2010 and to evaluate the outbreaks among MSM including all genotypes involved.

**Methods:**

All cases of acute hepatitis A among young adults notified to the Public Health Agency of Barcelona from 1989 to 2010 were included for analyses. We calculated the annual incidence rate and the incidence ratio male-to-female (M:F) as a marker for MSM. Spearman's coefficient was used to evaluate trends. We also evaluated the outbreaks among MSM and compared their characteristics using Chi-squared and ANOVA test. Fragment amplification of the VP1/P2A region was used for genetic analysis.

**Results:**

The median annual incidence for the period of study was 4.7/100000 among females and 11.7/100000 among males. The rate of hepatitis A for adult woman decreased over time (Spearman' coefficient = -0.63, *p *= 0.002), whereas there was no decrease for adult men (Spearman' coefficient = 0.097, *p *= 0.67). During the study period the M:F ratio increased (Spearman' coefficient = 0.73, *p *< 0.001).

Three large outbreaks among MSM were detected. When comparing outbreaks, there was a decrease in the percentage of bathhouse users (from 47% to 19%, *p *= 0.0001) and sex workers (from 6.5% to 0%) while the percentage of HIV infected individuals did not change significantly (range: 21%-28%, *p *= 0.36). The isolated strains were closely related to those circulating in Europe.

**Conclusions:**

Annual incidences remain high among MSM without tendency to decrease. More strategies which effectively reach the whole MSM community are needed.

## Background

Men who have sex with men (MSM) are a known group at risk for hepatitis A [1,2]. Periodic hepatitis A outbreaks among MSM have been reported since early 90's in Europe, United States, Canada, Australia and Spain [3-7]. The existence of an endemic population among MSM infected with the hepatitis A virus that sustains continuous circulation of particular strains and facilitates cyclical outbreaks has been postulated [8]. In Barcelona, hepatitis A immunization has been recommended for MSM since 1994. At the end of 1998, a vaccination campaign using a combined hepatitis A+B vaccine was started among all 12 year old pre-adolescents. In addition, a vaccination program in gay bathhouses began in 2004. Health care workers visited the bathhouses to offer information about hepatitis A, B, C and STIs, performed HIV rapid tests and administered vaccinations against hepatitis A and B free of charge. From 2004 to 2010, around 3000 sauna users were vaccinated (data from the Public Health Agency of Barcelona, 2010)

We described the incidence of hepatitis A in 20-44 year old adults from 1989 to 2010 in Barcelona, and evaluated the outbreaks among MSM describing their characteristics and the prevailing genotypes involved.

## Methods

### Study population and setting

We performed an observational study of all cases of hepatitis A residing in Barcelona reported to the surveillance system of the Public Health Agency of Barcelona (PHAB) between 1989 to 2010. Acute hepatitis A is a communicable disease that has been reported since 1986, physicians and laboratories in the city report cases to the PHAB. A case is defined by acute hepatitis symptoms combined with the presence of immunoglobulin M antibodies against hepatitis A virus (IgM anti-HAV). Since 1989 an electronic and confidential database of hepatitis A has been established.

Inclusion criteria: To avoid inclusion of age groups for which sexual activity is less likely to be related with disease acquisition only individuals aged between 20-44 were included for incidence rates calculation (these limits are based on the range of age of MSM involved in the outbreaks). For the same reason, cases with a known history of travel to an endemic area or contact with children were excluded.

### Data collection

Data collection is made routinely by the PHAB, for routing surveillance we use a standard questionnaire for hepatitis A of the Health Department of the Government of Catalonia. The questionnaire contains demographic data, information on the disease and risk exposures. However the information on sexual preferences not was included until 2002. For outbreak investigation we use the standard questionnaire and an annexed questionnaire designed for that purpose which also include: sexual condition, known contact with a case and sexual activity in the 6 weeks before symptom onset, HIV serological status, sexual work and bathhouse use. For the last outbreak, 2008, travel to industrialized countries during the six weeks preceding symptom onset was also collected.

The surveillance information is obtained by interview of the patient case or his/her doctor and also from clinical and laboratory records. Interviews are made by telephone by public health nurses from the PHAB.

### Outbreak investigation

We performed a description and comparison of the characteristics of all outbreaks detected by the surveillance system of the PHAB for the period of the analysis. For outbreak investigations, a case was defined as MSM, resident in Barcelona, presenting symptoms of acute hepatitis, and positive IgM anti-HAV. Cases that during the incubation period were in contact with an outbreak case were also considered as part of the outbreak, independently of whether they were MSM or not. All cases considered as part of an outbreak were included in the analysis. Also, molecular epidemiology was studied as part of the outbreak investigation.

### Genetic analysis

Serum from outbreak cases were sent to the Enteric Virus Laboratory of the Microbiology Department of the University of Barcelona for genetic analysis. Previously published primers [9], -3285 (5'AGTCACACCTCTCCAGGAAAACTT3'; reverse primer) and +2949 (5'TATTTGTCTGTCACAGAACAATCAG3'; forward primer) were used for the fragment amplification of the VP1X2A region containing an internal sequence of 168 bp (positions 3024 to 3191) that has been extensively used for genotyping. Sequencing of RT-PCR products was performed and multiple sequence alignments were performed using the ClustalW program (European Bioinformatics Institute).

### Statistical analysis

We calculated annual incidence rates of new cases of hepatitis A per 100000 inhabitants for males and females for the population aged between 20-44. Population data was based on the census published annually by the Statistics Department of the city [10]. Spearman's correlation coefficient was calculated to evaluate trends of rates. To determine sexual preference, we used the ratio male-to-female (M:F) as a surrogate marker for MSM, defined as the incidence of hepatitis A for male divided by the incidence for female. The M:F ratio is an indirect measurement for MSM which has been used when no direct data on sexual identity is available [11].

Categorical variables were presented as percentages, and continuous variables were presented as mean with corresponding range. The chi-squared test was used to compare categorical variables and F-ANOVA test for continuous variables. Statistical significance was established assuming an alpha error of 0.05. Foxpro was used to record data and SPSS software for Windows (version 18.0; SPSS, Chicago, IL) was used for analysis.

Patient consent was not needed as hepatitis A is a communicable disease. All data were treated in a strictly confidential manner following the ethical principles of the Helsinki Declaration of 1964 revised by the World Medical Organization in Tokio, 2008 and the Organic Law 15/1999 of Data Protection in Spain.

## Results

### Incidence and M:F ratio evolution

**Figure 1:**
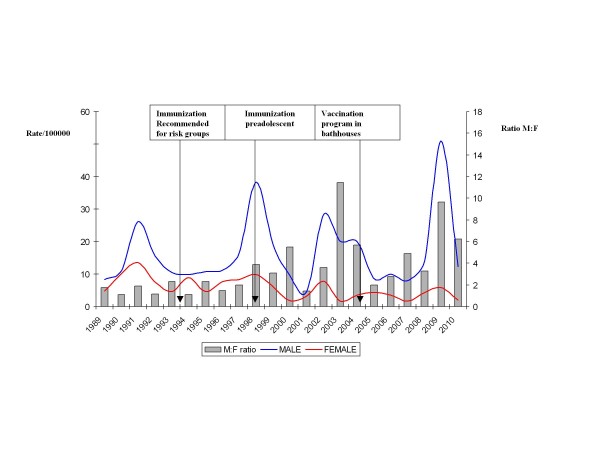
**Annual incidence of hepatitis A per 100000 inhabitants for males and females between 20 and 44 year old, and male-to-female ratio by years of symptom onset**. Barcelona, 1989-2010.

The median annual incidence was 4.7/100000 among adult females and 11.7/100000 among adult males for the period of study (Figure 1). The rate of hepatitis A for woman decreased over time (Spearman' coefficient = -0.60, *p *= 0.004), whereas no decrease was observed for men (Spearman' coefficient = 0.087, *p *= 0.7). During the study period men consistently showed higher rates than woman with cyclic peaks during outbreaks. Consequently, there was an increase in the M:F ratio (Spearman' coefficient = 0.70, *p *< 0.001).

### Characteristics of major outbreaks detected among MSM

Three outbreaks occurring in January-December 2002 (n = 48 cases), mid-2003 to mid-2004 (n = 60 cases), and in September 2008- June 2009 (n = 189 cases) were identified by the Surveillance System (Table 1). The mean (range) age was 31 (21-44), 31 (20-43) and 33 (19-58) year, (*p *= 0.10), for each outbreak respectively. The percentage of HIV-infected cases was 28%, 24% and 21% (*p *= 0.36), respectively. A significant decrease was observed among bathhouses users (47%, 40% and 19%, respectively) (*p *= 0.0001) and sex workers (6.5% in 2002 and no cases in 2009). During the last outbreak, 18% of cases had travelled abroad to industrialized countries during the six weeks preceding symptom onset.

**Table 1:** Characteristics of three outbreaks of hepatitis A among MSM in Barcelona

	January- December 2002	July 2003- July 2004	September 2008 - June 2009	*p*-value
Number of cases	48	60	189	-
Aged (mean, range)	31(21-44)	32 (20-43)	33 (19-58)	0.10

Men	47	60	185	0.71

Known Contact with a case	35%	18%	25%	0.31

Sexual activity in the 6 weeks previous to symptom onset	100%	NA	82%	< 0.0001

Bathhouses users	47%	40%	19%	< 0.001

Sex workers	6.5%	NA	0%	-

HIV infection	28%	24%	21%	0.36

Genotype	IB	IA	IA	-

*NA*: Not available

A total of 55 serum samples from outbreak cases were analyzed, one from the 2002 outbreak, 4 from the 2003-2002 outbreak and 50 from the 2008-2009 outbreak. The Genotype analysis of the 2002 outbreak showed the presence of genotype IB. In the 2003-2004 outbreak the isolated strain was genotype IA. In the 2008-2009 outbreak, strains belonging to genotype IA were isolated. This strain was unrelated to the previously isolated strains from the MSM outbreaks that occurred in Barcelona.

## Discussion

During the study period we observed an important reduction in the incidence of hepatitis A among adult women, however incidence among adult men has not changed substantially. This is translated in an increase in the ratio M:F. We also observed successive peaks in the M:F corresponding with outbreaks among MSM.

In our case, the vaccination recommendation in groups at risk and the program in gay bathhouses has not modified the incidence nor prevented new outbreaks. However, cases using bathhouses dramatically decreased in the last large outbreak, which could be explained by the vaccination program. Nonetheless, this intervention has had not impact among non bathhouse users and has not been enough to reduce the baseline incidence in the community. We expect that the vaccination campaign among preadolescent will have in the future an impact on the incidence of the diseases among young adults, when vaccinated individuals reach their twenties.

Hepatitis A among the MSM is a problem of international concern. The transmission of the infection across European borders caused by the same strains has been proved by genotypic analysis [8]. The strain circulating in the 2003-2004 outbreak was closely related to the cluster named MSM1 (Stene-Johansen, 2007) which was present among MSM in other European countries. Also, a strain indistinguishable from the one circulating in the last outbreak has been observed in sporadic cases among MSM in mainland England and Ireland [12]. In the last outbreak, a considerable proportion of cases had visited industrialized countries during the incubation period favoring disease transmission. In this context, the rapid exchange of information between countries when a large outbreak is detected and the international coordination of preventive interventions could be of great interest.

Our analysis is subjected to several limitations. Because the data is from cases reported to the surveillance system, results can be affected by under reporting. Sexual preference registered during outbreaks is self reported and its misclassification is possible. Sexual preference was not collected routinely by the Surveillance System until 2002, in our experience monitoring the M/F ratio is a useful tool when information on sexual preferences is not available. It seems possible that other existing outbreaks were not identified by the Surveillance System and we lack information about them. Regretfully, coverage of the hepatitis A among bathhouses users and among MSM population, that would have strengthened the idea of the relation between the decline of the proportion of bathhouses users in the last outbreak and the bathhouses campaign, could not be calculated as sound data on the number of bathhouses users among MSM population and general MSM population was not available.

## Conclusions

MSM present consistently high incidence without appearing to decrease. Prevention interventions which effectively reach the whole MSM community are needed.

## Competing interests

The authors declare that they have no competing interests.

## Authors' contributions

CT participated in the conception of the study, analysis and interpretation of data and drafting the manuscript, PO participated in the interpretation of data and critically revising the manuscript for intellectual content, ED participated in the conception of the study and critically revising the manuscript for intellectual content, R MP, AB, and UP carried out the genetic studies, MS made a substantial contribution in acquisition of data from the outbreaks, JAC participated in the conception of the study, interpretation of data and critical review of intellectual content and the Saunas Working Group made a substantial contribution in acquisition of data from bathhouses users. All authors have read and approved the final manuscript.

## Pre-publication history

The pre-publication history for this paper can be accessed here:

http://www.biomedcentral.com/1471-2334/12/11/prepub

## References

[B1] Centers for Diseases Control and Prevention Hepatitis A among homosexual men in United States, Canada, and Australia MMWR 1992 155 161 4 1741008

[B2] Urbanus AT van Houdt R van de Laar TJ Coutinho RA Viral hepatitis among men who have sex with men, epidemiology and public health consequences Euro Surveil 2009 14 1 5 http://www.eurosurveillance.org/ViewArticle.aspx?ArticleId=19421 10.2807/ese.14.47.19421-en 19941800

[B3] Centers for Diseases Control and Prevention Hepatitis A vaccination of men who have sex with men: Atlanta, Georgia, 1996-1997 MMWR 1998 47 708 11 9746398

[B4] Stokes ML Ferson MJ Young LC Outbreak of hepatitis A among homosexuals men in Sydney Am J Public Health 1997 87 2039 2041 10.2105/AJPH.87.12.2039 9431300 PMC1381253

[B5] Uhlmann S Buxton JA A provincial and territorial review of hepatitis A in men who have sex with men Can Commun Dis Rep 2007 33 1 11 18163239

[B6] Leentvaar-Kuijpers A Kool JL Veugelers PJ Coutinho RA van Griensven GJ An outbreak of hepatitis A among homosexual men in Amsterdam Int J Epidemiol 1995 24 218 222 10.1093/ije/24.1.218 7797346

[B7] Tortajada C de Olalla PG Pinto RM Bosch A Caylà J Outbreak of hepatitis A among who have sex with men in Barcelona, Spain, September 2008-March 2009 Euro Surveill 2009 14 3 5 http://www.eurosurveillance.org/ViewArticle.aspx?ArticleId=19175 19371516

[B8] Stene-Johansen K Tjon G Schreier E Bremer V Bruisten S Ngui SL Molecular Epidemiological studies show that hepatitis A virus is endemic among active homosexual men in Europe J Med Virol 2007 79 356 365 10.1002/jmv.20781 17311331

[B9] Robertson BH Averhoff F Cromeans TL Han X Khoprasert B Nainan OV Genetic relatedness of hepatitis A virus strains recovered from different geographical regions J Gen Virol 1992 73 1365 1377 10.1099/0022-1317-73-6-1365 1318940

[B10] Municipal statistics of Barcelona http://www.bcn.es/estadistica/catala/pub/index1.htm

[B11] Beltrami JF Shouse RL Blake PA Trends in infectious diseases and the male to female ratio: possible clues to changes in behavior among men who have sex with men AIDS Educ Prev 2005 17 6 Suppl B 49 59 16401182 10.1521/aeap.2005.17.Supplement_B.49

[B12] Sfetcu O Irvine N Ngui SL Hepatitis a outbreak predominantly affecting men who have sex with men in Northern Ireland, October 2008 to July 2009 Euro Surveill 2011 16 1 3 http://www.eurosurveillance.org/ViewArticle.aspx?ArticleId=19808 21392487

